# Spreading of soap bubbles on dry and wet surfaces

**DOI:** 10.1038/s41598-020-69919-7

**Published:** 2020-08-06

**Authors:** Patricia Pfeiffer, Claus-Dieter Ohl

**Affiliations:** Institute for Physics, Otto von Guericke University Magdeburg, Universitätsplatz 2, 39106 Magdeburg, Germany

**Keywords:** Fluid dynamics, Surfaces, interfaces and thin films

## Abstract

The spreading of soap bubbles after forming contact with a substrate is experimentally studied. We find for dry glass substrate that the rim of the spreading soap bubble follows the well known scaling law for inertia dominated spreading $r \sim t^{1/2}$ [Eggers, J., Lister, J., and Stone, H., J. Fluid Mech. 401, 293–310 (1999)]. Varying the viscosity of the soap solutions and the coating of the glass does not affect this spreading behavior qualitatively. Yet, on a wetted surface, the rim obtains a constant radial velocity. Here, the rim splits into two and this new rim trails the main rim. Interestingly, the central film enclosed by the two rims develops radially oriented wrinkles.

## Introduction

Through centuries soap bubbles have fascinated children and adults due to their beauty and fragility alike. Foams which are a dense packing of soap bubbles posses fundamental importance in many industrial processes such as waste water treatment^1^ or foam stability^2^. The fluid mechanics of their formation through coalescence has recently attracted more attention^3–5^. Besides the mutual interaction of soap bubbles to form foams, they are approaching and interacting with wet and dry container walls.

Let us first recall *liquid droplet* coalescence and their spreading as a closely connected fluid mechanical problem^6–9^. The coalescence of two closely spaced water droplets is initiated with the formation of a liquid bridge between the droplets^10^. Then the bridge growth $r_b$ was modeled by Eggers *et al.*^6^ to follow up to a logarithmic correction factor a linear growth, i. e. $r_b \sim t \, \text {ln}\,t$. In this regime, the growth of the liquid bridge depends on a competition between capillary forces driving the coalescence and viscous forces that oppose the merging^6, 7^.

In the inertia dominated regime (i. e. at Reynolds numbers $Re\gg 1$) Eggers *et al.* suggested the growth of the liquid bridge radius as $r_b \sim t^{1/2}$. The exponent results from balancing the kinetic energy and the interfacial stress, which is governed by the distance between the two droplets forming next to the liquid bridge^6, 8^.

For Reynolds number of $\approx 1$ a crossover between the inertial and the viscous regime occurs^7^. When two droplets come into contact, a capillary wave is generated. The duration of propagation of this wave across the drop determines the viscous time scale for droplet spreading^11^. Calculating the corresponding length and time scales at which inertia dominates (see Ref.^7^), one recognizes that it is rather difficult to observe the viscous regime for water-like fluids (length scale $\sim 15$ nm and time scale $\sim 10^{-10}$ s). Thus, to observe this regime a high viscosity fluid or low surface tension is needed.

The initial stage of droplet spreading on a smooth, flat surface is very similar to the coalescence of two droplets or the coalescence with a wet film. This can be attributed to the existence of a precursor film of typically one molecule in layer height, which is formed by van der Waals forces^9, 12^. The flow of the spreading droplet is driven due to symmetry away from the point of contact between droplet and substrate (i. e. in the perpendicular direction)^12, 13^. It spreads until it reaches a film thickness that is determined by van der Waals forces. On partially wetting surfaces the droplet spreads driven by capillarity as long as it has not reached its equilibrium contact angle^11, 12^. In the long-time limit the exponent of the spreading for a small, viscous droplet on a surface is 1/10, which was already predicted by Tanner^14^. Small means in this context smaller than the capillary length $l_c$: $r< l_c=\sqrt{\sigma /(\rho g)}$, with $\sigma$ being coefficient of surface tension, $\rho$ the density of the liquid and *g* the gravitational acceleration. In small droplets, surface tension plays the dominant role and the exponent can be determined by balancing surface energy and gravitational forces with the dissipation in the fluid^9, 13, 14^. For low viscosity droplets, the spreading dynamics is governed by the interplay between inertia and surface tension and a exponent of 1/2 was found in the early spreading stage^9, 12^ and 2/3 for later stages^9, 15^.

The cross-over time between the fast and the slow dynamics is determined by the droplet radius, the viscosity of the fluid, and the wettability of the surface^16^. The effect of the latter on the spreading of droplets has been studied: At early stages for partially wetting surfaces and low-viscosity droplets the power law was found to be independent of the surface wettability and only the pre-factor changes. Therefore, drops spread faster on a hydrophilic surface than on hydrophobic ^17^. In the inertia dominated regime of droplet spreading on partially wetting surfaces Bird *et al.*^11^ showed that $r\sim t^{1/2}$ does not hold anymore and the radius scales as $r\sim t^b$, where *b* decreases monotonously with increasing equilibrium contact angle ^11, 13^.

While spreading and coalescence of droplets is well understood, detailed studies of the contact dynamics of soap bubbles on dry and wet surfaces are scarce. Previous work is limited to experimental^3^ and numerical^18^ studies on partial coalescence of a soap bubble with a flat soap films. There the overall time for partial coalescence follows $t_p\sim R^{3/2}$, where *R* is the initial radius of the bubble.

This work aims to fill the knowledge gap to compare the spreading of soap bubbles on dry and wet substrates while varying film viscosity and their surface properties.

## Methods

A soap bubble is created by dipping a syringe into a soap solution and inflating the flat film by pressing the plunger, which results in a bubble filled with air attached to the tip of the syringe. The bubble diameters range between 10.0 and $12.8\pm 0.1$ mm. The ambient temperature is $22\pm 0.5\,^{\circ } \text{C}$. The syringe is pointing in the direction of gravity and is moved horizontally towards the substrate with a motorized translation stage (*Owis LTM 120-300-MiDS*). Thereby the surface of a glass substrate is approached with a constant speed of 0.5 mm/s. Upon touching the soap bubble spreads on the surface. The dynamics is illuminated with a collimated green laser ($\lambda = 532$ nm) and recorded with a high speed camera (Photron AX200) with 22,500 and 67,500 frames per second (exposure time is 1.1 $\upmu \hbox {s}$).

**Figure 1:**
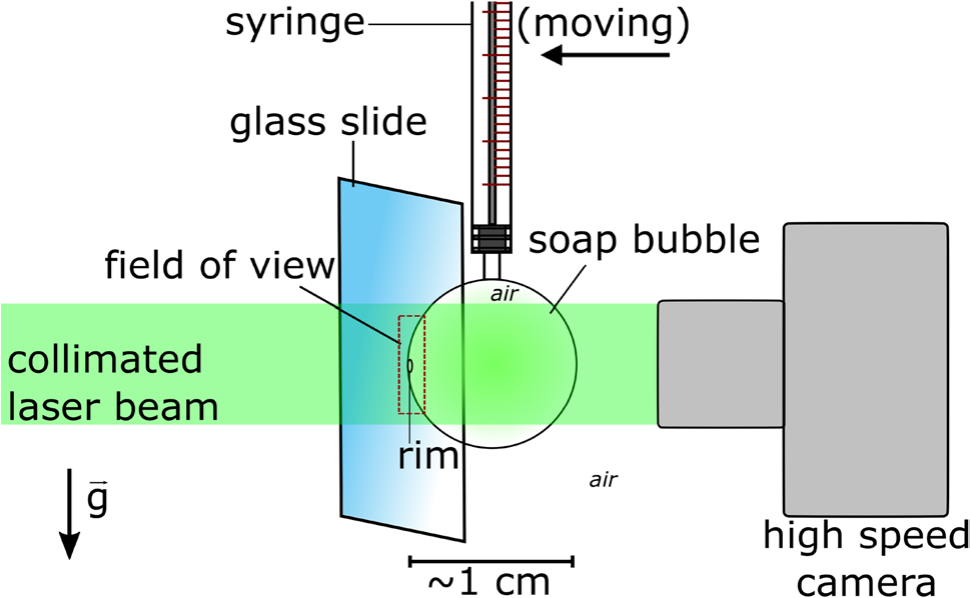
Experimental setup to study the spreading of soap bubbles on a glass substrate. The soap bubble is inflated with a syringe and translated slowly at constant speed towards the glass slide. The film spreading on the glass substrate is monitored with a high speed camera recording the interfacial film through the bubble.

The soap solution consists of deionized water and the anionic surfactant sodium dodecyl sulfate (SDS; *Sigma-Aldrich, BioXtra*$\ge 99.0\%$*(GC)*, CMC 7–10 mM (20–25 $^\circ \hbox {C}$)). To increase viscosity glycerol (Serva Analytical, grade 99%) is added to some of the water-surfactant solutions. We report experiments from three solutions with different viscosity and surfactant concentrations (cf. Table 1).

**Table 1:** Parameters of the three solutions in the preset study, i.e. concentration *c* of SDS and properties surface tension $\sigma$, dynamic viscosity $\eta$, and density $\rho$.

Solution	c(SDS)	Glycerol	$\sigma$	$\eta$	$\rho$
	[mM]	[wt%]	[mN/m]	[mPa s]	[$\hbox {kg/m}^3$]
1	10	0	$37\pm 2$	$1.01\pm 0.02$	$998\pm 2$
2	5	50	$46\pm 2$	$5.62\pm 0.06$	$1124\pm 2$
3	7.5	75	$51\pm 2$	$30.3\pm 0.9$	$1190\pm 2$

Four kinds of substrate coatings are used in the experiments to study the effect of the wetting properties of the aqueous surfactant solutions. These are indium tin oxide (ITO), a hydrophilic coating either with positive charges or without, and a hydrophobic coating.

To measure the static contact angle of the soap solutions on the glass slides, a small droplet (0.1 ml) was placed on the glass slide and a photograph was taken of the droplet after 20 s. The results are reported in Table 2. The contact angles vary slightly with placement of the droplet onto the surface. This is a common problem resulting from surface inhomogeneities, i. e. contact angle hysteresis^9^. Therefore we report in Table 2 not a single but a range of contact angles.

**Table 2:** Measured contact angle (liquid side) range $\Theta$ for droplets of the three different surfactant solutions S1, S2, and S3 (cf. Table 1) deposited on the on four different surfaces.

Glass	Company and type	$\Theta \hbox {(S1)}$ [$^\circ$]	$\Theta \hbox {(S2)}$ [$^\circ$]	$\Theta$(S3) [$^\circ$]
ITO	Being Tech Store, China	37–50	48–57	53–65
	Surface resistance $<10$ $\Omega /\hbox {sq}$			
Hydrophilic charged	Menzel GmbH Germany,	9–15	9–30	11–23
	Superfrost Plus			
Hydrophilic coated	Menzel GmbH Germany,	17–30	17–27	16–27
	Superfrost Excell Slide			
Hydrophobic	Paul Marienfeld GmbH Germany	57–69	68–84	72–86

The surface tension was measured with the pendant drop method and analyzed with a software using MATLAB (version R2019b, The Mathworks Inc.).

## Results

### Spreading on dry surfaces

**Figure 2:**
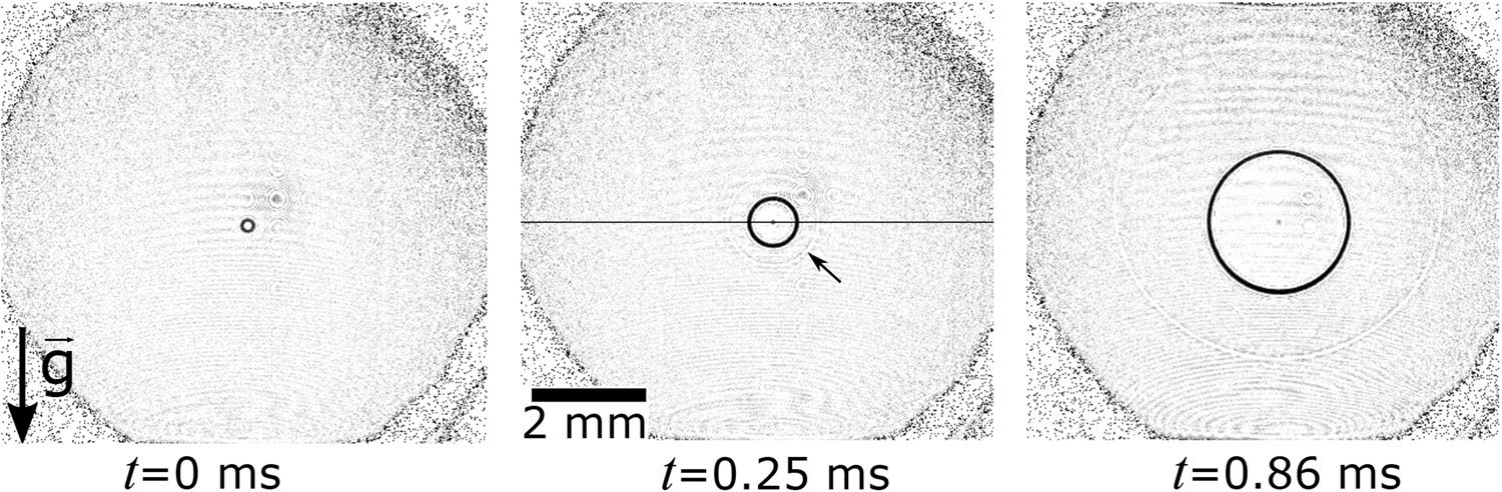
Images of the spreading rim of a soap bubble (solution S1) spreading on an ITO coated glass slide (black circle) with an initial radius of $R_0=5.0$ mm at different instants. The arrow points on the capillary wave, which precedes the rim, but travels on the bubble. The space–time plot shown in Fig. 3 is obtained at the location of the black horizontal line.

We report on the spreading of soap bubbles made from the three solutions (cf. Table 1) on four different glass plates. Figure 2 depicts exemplary three snapshots of the spreading dynamics once the soap bubble makes contact with the substrate. For this particular example the surface is coated with indium tin oxide (ITO). At time of contact, $t=0$, between the soap bubble and the substrate we see a small spot. This spot at $t=0.25\,\hbox {ms}$ has expanded into a ring of $\approx 0.2\,\hbox {mm}$ in diameter and keeps its nearly perfect round shape when growing to $\approx 2.2\,\hbox {mm}$ at $t=0.86\,\hbox {ms}$. Within the ring the image is brighter indicating a thinning of the liquid film, while the dark contrast of the rim is caused by accumulating liquid from the film into a highly curved region. Additionally, there is a second annular ring traveling with about twice the velocity of the rim indicated with an arrow in Fig. 2 at $t=0.25\,\hbox {ms}$. We identify this as a capillary wave originating from the initial point of contact. Interestingly, the capillary wave is traveling faster upwards than downwards. This behavior is explained with a thickness dependent capillary speed: due to gravity driven drainage the soap film is thinner above the initial point of contact than below, see Fig. 1.

**Figure 3:**
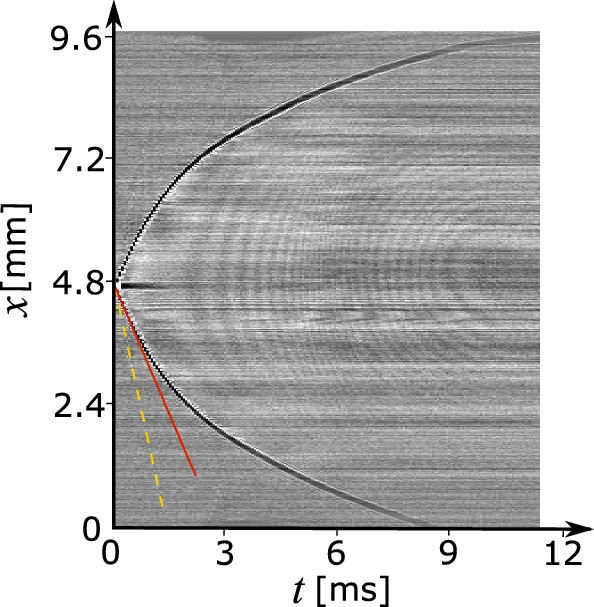
Space–time plot of the growth of the rim spreading over the ITO glass plate for solution S1. The initial velocity of the rim is $v=1.6\pm 0.2$ m/s, which is represented by the solid red line. The dashed line represents the capillary wave, which has a velocity of $3.6\pm 0.2$ m/s.

Next we focus on the velocity of the rim spreading on the glass substrate. Figure 3 is an artificial streak image generated from horizontal lines taken from consecutive images of the high-speed recordings. The pixel values are taken from the line indicated in Fig. 2 ($t=0.25\,\hbox {ms}$) just before contact until $t=11\,\hbox {ms}$. The initial radius of the soap bubble is $5.0\pm 0.1$ mm. The tangent of the $x-t$ line is the velocity of the spreading rim. After contact at $t=0$ the rim grows with a constant velocity $v=1.6\pm 0.2$ m/s, which is indicated by the red line. After about $t_0\approx 0.2\pm 0.05\,\hbox {ms}$ the rim velocity decelerates and eventually approaches 0. That is the moment when the bubble adopts a hemispherical shape on the glass slide, i. e. $2^{1/3}$ times the diameter of the original bubble. We call this time $t_0$ the cross-over time.

The velocity of the capillary wave $v_c$ can be determined from the slope of the dashed line in the $x-t$ plot (Fig. 3). The point of contact between bubble and glass plate was selected and another one on the line produced by the capillary wave, where it appears still linear. Between these points a line was calculated to obtain the spatial positions of the capillary wave over time, from which the velocity can be calculated. To verify this method, the position of the capillary wave is tracked in each frame and the velocity is determined from the position over time. Since we only observe a projection of the wave on a plane, the curvature of the bubble needs to be taken into account. The spatial distance *y* needs to be corrected: $y_{corrected}=r(\pi /2-\text {arccos}(y/r))$, *r* is the initial radius of the bubble. The horizontal speed of the capillary wave is $3.6\pm 0.2$ m/s. The knowledge of the velocity of the capillary wave allows us to calculate the film thickness of the bubble. The wave speed of an anti-symmetric capillary wave $v_c$ can be calculated as^19, 20^

1 $$\begin{aligned} v_c=\sqrt{\frac{2 \sigma }{\rho h}}, \end{aligned}$$

where $\sigma$ is the surface tension, $\rho$ is the density of the soap solution, and *h* is the film thickness. Thus, the film thickness of the soap bubble can be calculated from the velocity of the capillary wave in the horizontal direction, which is $5.7\pm 0.4\,\upmu \hbox {m}$.

**Figure 4:**
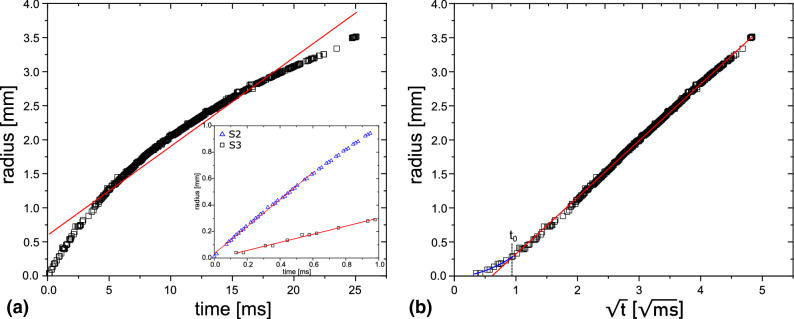
Evaluation of the spreading law exemplary shown for a bubble of solution S3 with $R_0=6.4$ mm on ITO glass: (**a**) *r* over *t* and (**b**) *r* over $\sqrt{t}$. The inset in (**a**) shows the early time behavior of the spreading for solutions S2 (blue triangles) and S3 (black squares). The cross-over time $t_0$ is the intersection between the fits of both parts of the curve. The fit of the blue curve is $r=a\,t$ and the red curve follows $r=b + c \sqrt{t}$.

From the space–time plot we extract the spatial coordinate of the rim. Let us first compare the measurements with a power law of for the early spreading of a droplet on a dry surface, i. e. $r\sim t^{1/2}$^12^. In Fig. 4 the rim radius *r* is plotted in (a) as a function of *t* and in (b) as a function of $\sqrt{t}$. Comparing both graphs, we see that the spreading follows very accurately a $r\sim t^{1/2}$ dependency, except during the very early time: here $t< 0.8\,\hbox {ms}$.The cross-over time $t_0$ is determined from (b), where the first part (blue curve) is fitted with $r=a\,t$ and the remaining data are fitted with $r=b+c\sqrt{t}$ (*a*, *b* and *c* are fitting parameters). The intersection of both fits gives $t_0$.

**Figure 5:**
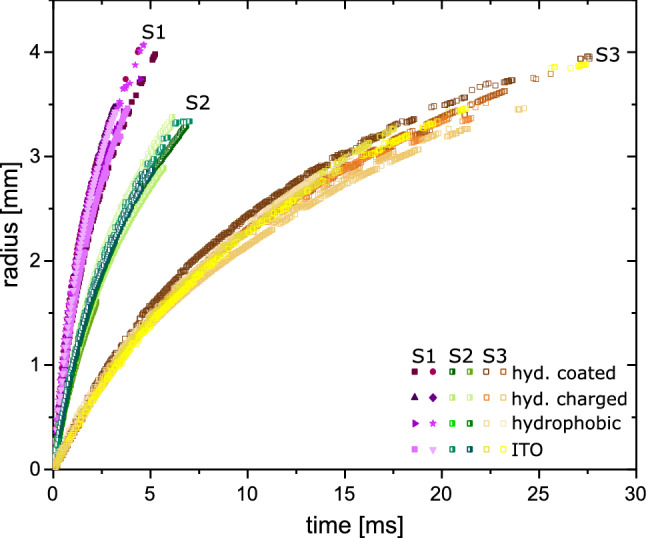
Spreading of different soap bubbles with an initial radius of $R_0=6.4\pm 0.1$ mm made from three different soap solutions S1, S2 and S3 (cf. Table 1).

Next the effect of viscosity is investigated by varying the amount of glycerol added to the solution (cf. Table 1). In Fig. 5 the location of the rim *r* as a function of time for three different solutions are shown. Additionally, four different glass substrates are used. The soap solutions show different equilibrium contact angles on the different substrates. The values are presented in Table 2. However, these different contact angles have no significant effect on the spreading of the soap bubble. Small deviations in the spreading can be attributed to a varying film thickness of the soap bubble. The soap solution S1 without glycerol and lowest viscosity is spreading the fastest. For S2 and S3 the spreading event lasts longer, which can be seen in Fig. 5. Additionally, the cross-over time when deceleration starts is affected by the viscosity: solution S2 has an cross-over time at $t_0=0.30\pm 0.05$ ms ($v=0.62\pm 0.05$ m/s) and solution S3 of $t_0=0.6\pm 0.1$ ms ($v=0.32\pm 0.05$ m/s).

**Figure 6:**
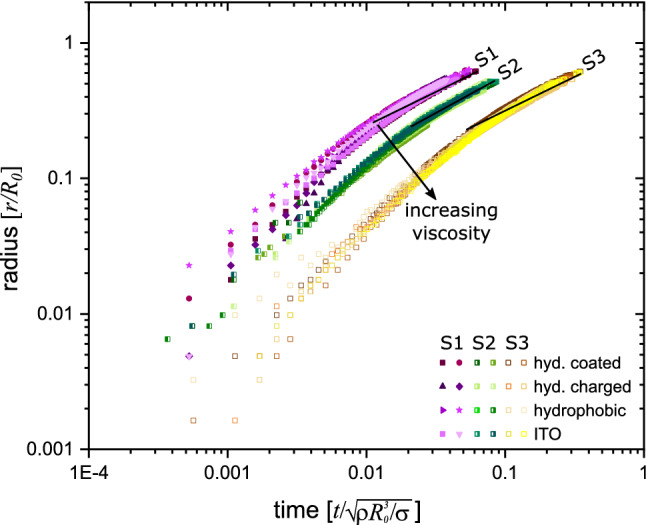
Comparison of the spreading of a soap bubble made from three different solutions on a glass substrate in dimensionless units. The radius *r* is divided by the initial radius $R_0$ of the bubble and time is scaled by $\sqrt{\rho R_0^3/\sigma }$. The black lines is a least square fit $r=a+b^{0.5}$ to the measurements.

The final radius the rim attains is determined by the radius of the bubble before contact. Typically, the rim grows to about the size of the soap bubble just prior contact. As this initial size varies between experimental runs we re-scale the radius of the rim with the initial radius of the bubble $R_0$. Accounting for balance of inertia and capillary forces, the time is scaled by the capillary-inertial time $t'=\sqrt{\rho R_0^3/\sigma }$  ^8^. Interestingly Fig. 6 reveals that after this rescaling of time and space the $r-t$ curves for an particular soap solution collapse into a single curve. With higher viscosity the curves are shifted to the right; thus their rims are slower than that of the pure soap solution (S1). Yet, we observe a considerable spread in the velocity of the radius during the early times. We attribute this to a variation of the film thicknesses of the soap bubbles which is not controlled in the experiments. Here, a thicker film reduces the initial acceleration due to inertia of the forming rim^5^. Thus thicker films are initially slower than thinner films explaining the spread of velocities.

### Spreading on wet surfaces

Next we report and discuss the spreading of soap bubbles on wetted surfaces.Note that only the macroscopic wetting is studied here. The wetting according to the molecular-kinetic theory^21^ cannot be resolved in the current setup. The wetting is realized with hydrophilic glass slides that are wetted with a glycerol containing solution (S2 or S3). This coating is obtained by spreading a bubble on the dry surface prior to the present experiment. Solution S1 shows dewetting and is therefore not used as a wet coating. Solutions S2 and S3 form a thin stable fluid layer with an almost uniform thickness of approx. 50 $\upmu \hbox {m}$. When a soap bubble is approaching those wetted glass slides, the spreading characteristics are altered considerably as compared to the dry surface. We now observe a rim that grows linear with time.

**Figure 7:**
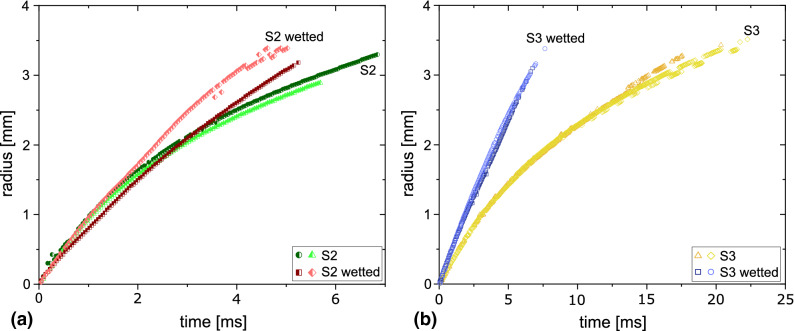
Comparison of the spreading of a soap bubbles (**a**) made from S2 and (**b**) S3 on a hydrophilic surface, which is either dry or perfectly wetted.

Figure 7 compares the rim growth for solutions S2 and S3 on a dry and on a wetted hydrophilic surface. The spreading of solutions S2 and S3 on a dry surface are colored in green and yellow, respectively, and the spreading on the wetted surface is colored in red and blue for S2 and S3. Interestingly, for S2 the difference with the non-wetted surface is small until $t=2.5\,\hbox {ms}$. The linear slope of these curves is almost identical for all surfaces: $\approx 0.62\pm 0.2\,\hbox {m/s}$. The rim growth on the wetted surface remains linear with time before its speed decreases once the radius becomes comparable to the equilibrium radius of the hemispherical bubble. For solution S3, the spreading also remains constant on the wetted surface, the spreading velocity is $0.45\pm 0.05$ m/s. This, however, is considerably faster than the spreading on a *dry surface* (i. e. $0.32\pm 0.05$ m/s) due to the lubricating effect of the liquid layer.

**Figure 8:**
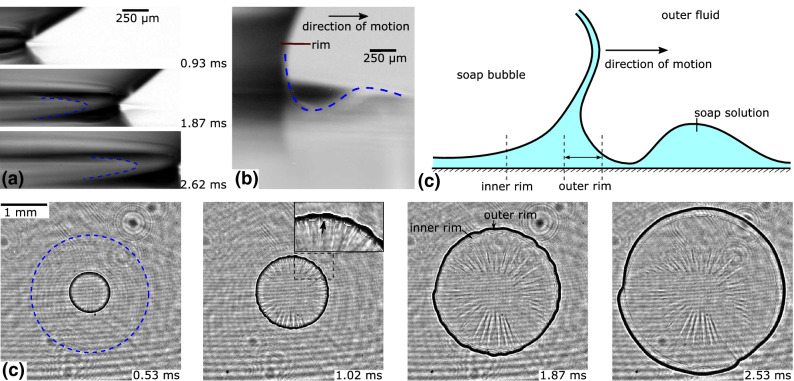
Spreading of a soap bubble on a wetted surface. (**a**) Side view: The merging of the bubble occurred outside the field of view on the left side of the image and the rim propagates to the right. The dashed line indicated the position of the second rim. (**b**) Inclined side view of the spreading rim, which forms a valley and a hill of liquid in front of it. The dashed line indicated the height profile of the liquid. (**c**) Schematic drawing of the side view of the spreading rim. (**d**) Top view of the spreading under monochromatic illumination. The dashed blue circle circles the area where interference fringes occur due to thickness variations of the fluid layer. The arrow in the inset at $t=1.02\,\text{ms}$ points on the evolving second rim.

Observing the spreading in a top view, the formation of a second rim can be seen (see arrow in Fig. 8d at $t=0.02$  ms). This rim is thinner and moves a little slower than the leading rim. The latter displays undulations, which occur approx. 1 ms after the formation of the liquid bridge (see inset). In order to understand the origin of the second rim, experiments are performed from the side view (Fig. 8a). Here it can be seen that behind the leading rim a wedge shaped soap film follows. Its tip is marked with a dashed line in (a). Between this line and the leading rim the inner rim appears in the interferometric measurements. Since the outer rim collects more and more fluid from the substrate, the volume of the wedged shaped fluid film increases, such that it becomes wider (the distance between the leading rim and the dashed line in (a) increases). The inclined side view (Fig. 8b) helps to understand the geometry. Here, the outer rim builds up a crest of fluid upstream. These thickness variations are visible under monochromatic illumination, since interference fringes occur in front of the rim (dashed circle in (d)). The entire geometry is sketched in (c) and the positions of the inner and the outer rim are marked. Note that in the side view the glass slide is now oriented horizontally, such that the thin film coating does not run off the glass slide. Hence, the thickness of the fluid film is thicker than in the top view experiments. In the latter, the film thickness on the glass slide lies around 50 $\upmu \hbox {m}$, whereas it is around 200 $\upmu \hbox {m}$ for the side view experiments.

Additionally to the formation of the inner and the outer rim, an instability of the rim was observed in some of the experiments, which is characterized by equally spaced indentations of the outer rim (cf. Fig. 8d). The wavelength of these indentations grow with time due to the radial expansion of the rim. Furthermore, radial bright and dark stripes can be seen downstream of the rim. We hypothesize that these may be the result of an instability during the formation and early expansion of the rim upon splitting.

The rim expands slower in regions with a thicker film. This is upon close inspection where brighter radial wrinkles connect to the rim, see Fig. 8d at $t=1.02$ ms. At the endpoints of the darker radial rays the rim has already split into two. If we further assume that the surface curvature of the film refracts the collimated light, we expect brighter regions close to the crests and darker regions at the troughs.

For the troughs the film profile looks like as sketched in Fig. 8c, where the inner rim appears at a certain curvature at the wedge shaped area behind the leading rim. However, when crests are present, the curvature changes which reduces the contrast of the inner rim.

## Discussion

The experiments were conducted to reveal the spreading dynamics of soap bubbles on rigid and wet substrates. The power law derived by Eggers *et al.*^6^ for the coalescence of droplets provides a seemingly good fit to the spreading of soap bubbles, too. At least for the parameter regimes covered in the present study.

Right within the experimental temporal and spatial resolution of 14.8 $\upmu \hbox {s}$ and 22 $\upmu \hbox {m/pixel}$ we observe a linear growth of the rim, i. e. at a constant velocity. After some cross-over time, the rim grows with the square root of time, i. e. the velocity drops with power −1/2. The value of the cross-over time depends on the viscosity of the fluid: with increasing viscosity the cross-over time increases as well. An exponent of 1/2 in the power law has previously been observed for inertia dominated spreading droplets on a surface^12^. When soap bubbles spread in a surface the rim becomes thicker since it is collecting excess liquid from the bubble. The drainage and the stability of the water film are governed by intermolecular forces and surface rheology. In the presence of surface active molecules the drainage is strongly affected by a gradient in surface tension (Marangoni effect)^22^. The contribution of surface active molecules should be included in a model to account their importance.

The spreading velocities for the three solutions before the cross-over time were determined from the space–time plots (cf. red line in Fig. 3). They are $1.6\pm 0.2$ m/s, $0.62\pm 0.05$ m/s and $0.32\pm 0.05$ m/s for solutions S1, S2 and S3, respectively. These velocities decrease with increasing viscosity of the solutions as expected. Also the velocities of the capillary waves in the three solutions vary: Solution S1 shows a mean velocity of $v_c\approx$ 3.5 m/s (film thickness $h=6.2\,\upmu \hbox {m}$). In solution S2 the capillary wave has a mean velocity of $v_c\approx$ 2.4 m/s (film thickness $h=13.6\,\upmu \hbox {m}$), and in solution S3 it is $v_c\approx$ 1.9 m/s (film thickness $h=23.3\,\upmu \hbox {m}$). This is not unexpected as it depends on the density and the surface tension, which vary for all three solutions.

In Fig. 2 the capillary wave has different velocities towards the top and the bottom of the bubble. This is caused by a varying film thickness. Due to gravity, the film is thicker at the lower pole of the bubble than at the upper one. Tracking the position of the capillary wave either towards the top and the bottom of the bubble we obtain a film thickness towards the bottom of approximately $15\pm 1\,\upmu \hbox {m}$ which decreases to the top to 2$\pm 1\,\upmu \hbox {m}$.

Figure 6 depicts the scaled rim vs. time curve. After a cross over time $t_0$, the curves from a single solutions collapse onto a single curve. The deviation at the early time is caused by slight differences in the film thickness.

On wet surfaces we observe marked differences when the bubble contacts and spreads compared to a dry surface. The radius grows linearly right from the beginning and within our experimental resolution. The rim only slows down when it reaches the radius of the soap bubble. We explain this behavior with a purely viscous spreading^7, 11^ where inertia does not play a role.

Additionally, the formation of an instability is observed during the spreading on a wetted surface. We find the formation of radial oriented stripes trailing the rim and explain it with height undulations of film downstream of the rim.

To understand if acceleration may cause this instability we estimate its magnitude upon contact from the Laplace pressure

2 $$\begin{aligned} G=\frac{\text {D}u}{\text {D}t}=-\frac{1}{\rho } \frac{2 \sigma }{R_0}\quad , \end{aligned}$$

where *u* is the velocity of the fluid particle in the rim, *t* is the time, $\rho$ is the density of the liquid and $\sigma$ is the surface tension. We obtain typical values for the acceleration *G* of $-12$ $\hbox {mm/s}^2$ with $\sigma =0.037$ N/m, $\rho =10^{3}$ $\hbox {kg/m}^3$. This acceleration of a fluid particle is also achieved, when assuming a circle, which is driven against a flat surface with a velocity of 0.5 mm/s. The horizontal acceleration of a particle, which is located on the top of the circle moving towards one side is approx. $-8$ $\hbox {mm/s}^2$. With this small acceleration of the fluid, a Rayleigh-Taylor instability, which occurs during the coalescence of two bubbles ^5^ can be excluded. Instead we speculate that the wrinkle formation is caused by a Marangoni flow. The wrinkles remind of stripes evolving in evaporating films on an inclined surface, such as wine in a glass^23^. Similar structures were found by Wodlei *et al.*^24^, during evaporation of an oil droplet containing a surfactant, which is placed on a soap solution. The authors connected the number of wrinkles to the relation of a critical layer height, below which wrinkles can occur and the undisturbed film thickness at the edge of the drop. The prediction of such a critical height by Wodlei *et al.*^24^ could explain, why in our experiments wrinkles are not present in every experiment, but in only 50% of the cases.

In this work, the spreading of soap bubbles on different substrates is studied. The spreading of the contact line was found to follow the power law $r\sim t^{1/2}$ almost appropriate for the inertia dominated regime. At very early times ($t \le 0.2$ ms for low viscosity soap bubbles), a deviation from this law can be observed. A purely linear increase of the radius with time was found. At late times, the radius approaches a constant size, since the bubble approaches its equilibrium shape, a hemispherical soap bubble on the glass substrate. A confirmation of the power law $r\sim t^{1/2}$ was lacking so far for soap bubbles. The spreading velocity depends on the viscosity of the fluid, but not on the equilibrium contact angle between soap solution and glass. On wetted surfaces, a linear growth of the rim was found, which can be attributed to a purely viscous spreading. During the spreading, a second rim emerges and trails the main outer rim. It becomes visible because of its wedge-shape falling off from the outer rim. At a certain position, total refraction of the laser light occurs, which results in a thin dark ring (inner rim) behind the leading outer rim. Additionally, the formation of wrinkles can be observed, when a certain film thickness on the glass substrate exists. They may be formed due to a Marangoni flow.
